# Application of loop-mediated isothermal amplification combined with colorimetric and lateral flow dipstick visualization as the potential point-of-care testing for *Corynebacterium diphtheriae*

**DOI:** 10.1186/s12879-020-05037-z

**Published:** 2020-04-25

**Authors:** Aleksandra A. Zasada, Aldona Wiatrzyk, Urszula Czajka, Klaudia Brodzik, Kamila Formińska, Ewa Mosiej, Marta Prygiel, Katarzyna Krysztopa-Grzybowska, Karol Wdowiak

**Affiliations:** Department of Sera and Vaccines Evaluation, National Institute of Public Health – National Institute of Hygiene, Chocimska 24, 00-791 Warsaw, Poland

**Keywords:** LAMP, POCT, Diphtheria, *Corynebacterium diphtheriae*, Colorimetric detection, Lateral flow dipstick

## Abstract

**Background:**

Diphtheria outbreaks occurred in endemic areas and imported and indigenous cases are reported in UE/EEA. Because of the high infectiveness and severity of the disease, early and accurate diagnosis of each suspected case is essential for the treatment and management of the case and close contacts.

The aim of the study was to establish simple and rapid testing methods based on Loop-Mediated Isothermal Amplification (LAMP) assay for the detection of *Corynebacterium diphtheriae* and differentiation between toxigenic and non-toxigenic strains.

**Methods:**

*Corynebacterium diphtheriae* and *Corynebacterium ulcerans* isolates from the National Institute of Public Health-National Institute of Hygiene collection were used for the development of LAMP assay for the diagnosis of diphtheria and nontoxigenic *C. diphtheriae* infections. Various colorimetric methods for visualization of results were investigated. Sensitivity and specificity of the assay were examined using a collection of DNA samples from various gram-positive and gram-negative bacteria.

**Results:**

The LAMP assay for *tox* and *dtxR* genes was developed. The sensitivity and specificity of the assay were calculated as 100%. The detection limit was estimated as 1.42 pg/μl concentration of DNA template when the reaction was conducted for 60 min. However, the detection limit was lowered 10 times for every 10 min of reduction in the time of incubation during the reaction. Positive results were successfully detected colorimetrically using hydroxynaphthol blue, calcein, QuantiFluor, and lateral flow Milenia HybriDetect dipsticks.

**Conclusion:**

The assay developed in the study might be applied for point-of-care testing of diphtheria and other *C. diphtheriae* infections as well as for other infections caused by diphtheria-toxin producing *Corynebacterium* species. It is highly sensitive, specific, inexpensive, easy to use, and suitable for low-resource settings.

## Background

Diphtheria is an acute, highly infectious and potentially lethal infectious disease of humans. The disease is caused by diphtheria toxin-producing strains of *Corynebacterium diphtheriae, Corynebacterium ulcerans,* and *Corynebacterium pseudotuberculosis.* The infection can be transmitted through contact with infected persons and objects that are touched by them. The disease could be presented as respiratory or cutaneous diphtheria, depending on the anatomic site that is affected by the toxigenic corynebacteria. Rarely other sites can also be affected such as ear, eye, and vulva. Diphtheria toxin absorbed from the mucosal or cutaneous lesions causes toxic damage to the nervous system, myocardium and kidneys. In respiratory diphtheria cases, formed pseudomembranes can cause obstruction in the airways [1].

The infections caused by toxigenic corynebacteria seemed to be well controlled in developed countries since the introduction of vaccination against diphtheria in the 1940s. But, infections recorded during the last several years point at *C. diphtheriae* and *C. ulcerans* as reemerging human pathogens. According to ECDC data, the number of confirmed diphtheria cases in EU/EEA increased over three times from 2011 to 2015 [2]. Domestic pets and other animals have been described as novel reservoirs and sources of diphtheria infection [3–5]. Moreover, diphtheria is endemic in many countries in Asia, the South Pacific, the Middle East, and Eastern Europe and in Haiti and the Dominican Republic; outbreaks in Indonesia, Thailand, Laos, South Africa, Sudan, and Pakistan have occurred since 2011 [6]. According to WHO data, 28,358 cases of diphtheria were recorded between 2012 and 2016 worldwide. In the period 2012–2016, India had the largest total number of reported cases each year, with a 5-year total of 17,497 cases, followed by Indonesia and Madagascar with 2739 and 4492 reported cases, respectively [7]. In addition, the diphtheria cases were described in asylum seekers in Europe [8, 9]. In Poland, the last diphtheria case was recorded in 2000. But in 2004 the first case of sepsis caused by nontoxigenic *C. diphtheriae* was recorded and since then increasing number of nontoxigenic *C. diphtheriae* infections, including invasive infections, has been recorded every year [10, 11].

Because of the high infectiveness and severity of the disease, early and accurate diagnosis of each suspected case is essential for the treatment and management of the case and close contacts. Rapid microbiological tests are of high value because clinical diagnosis is not easy and might be confused with other causes, such as streptococcal sore throat or tonsillitis [12]. Misdiagnosis is the high risk particularly in countries where the diphtheria is uncommon. Gap analysis on securing diphtheria diagnostic capacity in the EU/EEA revealed that 3% of EU Member States have minimum or no diagnostic capability and 17% have only partial diagnostic capability [12]. Point-of-care diphtheria testing is especially important in refugee camps and developing countries, where access to medical laboratories is extremely limited as well as in the investigation of an infection source.

Our study aimed to establish simple and rapid testing methods based on LAMP assay for the detection of *C. diphtheriae* and differentiation between toxigenic and non-toxigenic strains as well as detection of other infections caused by diphtheria-toxin producing *Corynebacterium* species. Additionally, we compared various methods for visualization of amplified products. We decided to apply LAMP technology because it enables efficient DNA amplification under isothermal conditions. To find the optimal method for amplicon detection we compared commercially available lateral flow dipsticks and cheaper colorimetric methods with various dyes.

## Methods

### Bacterial strains and DNA extraction

A total of 51 bacterial strains were used in the study, including 9 toxigenic and 31 nontoxigenic *Corynebacterium* strains and 11 strains of other bacterial species that might be present in the respiratory tract. The toxigenic strains included 5 *C. diphtheriae* clinical isolates, one *C. ulcerans* clinical isolate, and reference *C. diphtheriae* strains such as PW8, NCTC 10648, and NCTC 3984. The nontoxigenic strains included 30 *C. diphtheriae* clinical isolates and the reference *C. diphtheriae* strain NCTC 10356. Other bacterial species that might be present in the respiratory tract included *Streptococcus salivarius*, *Streptococcus pneumoniae*, *Streptococcus pyogenes*, *Streptococcus epidermidis*, *Haemophilus influenzae*, *Moraxella catarrhalis*, *Staphylococcus aureus*, *Corynebacterium pseudodiphtheriticum*, *Pseudomonas aeruginosa*, *Escherichia coli*, and *Klebsiella pneumoniae*.

DNA extraction was performed for 24 h bacterial cultures on the medium appropriate for the bacterial species by using DNeazy Blood and Tissue Kit (Qiagen, Germany) according to the manufacturer’s instructions for Gram-positive and Gram-negative bacteria, respectively.

### Bacteria identification

*Corynebacterium* strains were identified and biotyped based on the colony morphology on tellurite agar plates and ApiCoryne tests (Biomerieux, France). Toxigenicity was tested using Elek test. Additionally, the presence of *tox* gene was verify by PCR, according to WHO Manual for Laboratory Diagnosis of Diphtheria [13].

Other non-Corynebacterium strains mentioned above were identified using conventional microbiology methods, including appropriate selective media, Gram-staining and biochemical tests.

### Loop-mediated isothermal amplification (LAMP)

LAMP primer sets for the detection of *tox* gene coding diphtheria toxin and *dtxR* gene coding global regulator were designed by using LAMP designer software PrimerExplorer V4 (https://primerexplorer.jp/e/) based on the nucleotide sequence of the *C. diphtheriae* NCTC 13129 whole genome, available from GenBank under the number BX248353. Each LAMP primer set included two outer (F3, B3), two inner (FIB, BIP), and two loop primers (LF, LB). The sequences of the oligonucleotide primer sets used in the study are presented in Table 1. For the detection of amplified products using the lateral flow dipsticks, the FIB and BIP primers were labeled with biotin and fluorescein isothiocyanate (FITC), respectively, at the 5′ end. For the colorimetric detection of amplified products, unmodified primers were used. Modified primers were obtained from Metabion (Germany) and unmodified primers were obtained from Genomed (Poland). LAMP was carried out in a final reaction volume of 25 μl. The concentration of primers in the reaction mixture was optimized for each target individually. Finally, the reaction mixture for both targets contained 0.8 μM of FIB and BIP primers each, 0.2 μM of F3 and B3 primers each, 0.4 μM of LF and LB primers each, 1× reaction buffer containing 20 mM Tris-HCl, 50 mM KCl, 10 mM (NH_4_)_2_SO_4_, 2 mM MgSO_4_, 0.1% Tween 20 (New England Biolabs, USA), 0.2 mM dNTP (Sigma-Aldrich, USA), 0.2 M betaine (Sigma-Aldrich, USA), 8 units Bst 2.0 DNA Polymerase (New England Biolabs, USA), and 2 μl sample DNA. The reaction was optimized at the temperature ranging from 62 °C to 70 °C and conducted for 60 min. During the optimization step, the results of the reaction were analyzed using agarose gel electrophoresis.

**Table 1:** LAMP primers used in the study

Target gene	Name	Sequence (5′ → 3′)
*tox*	LF-toxIII	GCATAGTTAGCCCCAGCGAAT
	LB-toxIII	ACTTCCTGGTATCGGTAGCGT
	F3-toxIII	CGGCATTAGAGCATCCTG
	B3-toxIII	CTAGCTCTCCTACCAATGGA
	FIP-toxIII	CGCAACGTTTACTGCCCATTTTCTTACTGGGACCAATCCTGT
	BIP-toxIII	AAGACAACTGCTGCTCTTTTTTTCGATATTGTGGTGAACGGCAC
*dtxR*	LF-dtxRIII	TCGTCACTCATAACGTGTTCC
	LB-dtxRIII	CGGCGTAGGCAATTCTGA
	F3-dtxRIII	AACATCGCTTAGCTGAGC
	B3-dtxRIII	CGTTAATCTGAACAATGCGTAC
	FIP-dtxRIII	TTCACGAGCCTGCGTTCTTTTAAAAGTTCACGATGAAGCCTG
	BIP-dtxRIII	CAATTCCAGGTCTCGACTTTTGAACTTCAATAACGCGAGTTCCG

### Detection of product amplification with the lateral flow dipsticks

Milenia HybriDetect dipsticks (Milenia Biotec, Germany) were used for the detection of the amplified products labeled with biotin and FITC. Ten microliters of the reaction mixture were pipetted directly on the sample application area on the dipstick. Then, the dipstick was placed with the same application area into 100 μl of HybriDetect assay buffer and incubated for 5–15 min in an upright position. The results were regarded as positive when two bands were visible (a control band and a test band) or as negative when only a control band was visible.

### Colorimetric detection of amplified products

For the colorimetric detection of amplified products, 5 indicators were used comparatively: neutral red, phenol red, hydroxynaphthol blue (HNB), calcein and QuantiFluor. Neutral red and phenol red are pH indicators. They are added to the pre-reaction solution. The progress of LAMP reaction is related to lowering of the solution pH, which can be observed directly as color change of faint orange to pink (neutral red) or red to yellow (phenol red) [14]. Hydroxynaphthol blue and calcein are metal ion indicators. They are also added to the pre-reaction solution. When Mg^2+^ ion concentration decreases in the progress of LAMP reaction, the color change of the indicators can be observed directly [15]. The color shift is violet to blue for HNB and orange to fluorescent green for calcein. QuantiFluor is a DNA intercalating dye. It is added to the solution after the reaction is completed. When the LAMP reaction is positive, a color change of orange to fluorescent yellow is observed under ambient light condition.

Neutral red (Sigma-Aldrich, USA) and phenol red (Sigma-Aldrich, USA) were dissolved in deionized water or 1 M NaOH, respectively, at 50 mM to prepare a stock solution and diluted to 2.5 mM. For the optimization of the concentration of the indicators in the reaction solution, the following final concentrations were tested: 0.2, 0.15, 0.1, 0.05, and 0.01 mM. HNB was dissolved in deionized water at 50 mM to prepare a stock solution. Then, the solution was diluted and tested in the LAMP reaction at the following final concentrations: 1, 0.5, 0.32, 0.25, 0.16, 0.125, 0.08, and 0.04 mM. The calcein stock solution consisted of 0.5 mM calcein (Novazym, Poland) and 10 mM MnCl_2_ (Sigma-Aldrich, USA) in deionized water. To select an optimal concentration, the following volumes of the stock solution were added to the reaction solution: 0.25, 0.5, 1, 1.5, and 2 μl. The amount of QuantiFluor (Promega, Germany) in the post-reaction solution was optimized by the addition of the following volumes of the dye: 2, 1, and 0.5 μl.

### Determination of specificity, sensitivity, detection limit, and minimal reaction time

Specificity and sensitivity of the LAMP were investigated using abovementioned bacterial species that can be present in respiratory tracts. The sensitivity was calculated as follows: A/(A + C) × 100%, and the specificity was calculated as follows: D/(B + D) × 100%, where A is the number of true positive results, B is the number of false-positive results, C is the number of false-negative results, and D is the number of true negative results. Additionally, positive (PPV) and negative predictive value (NPV) were calculated as follows: PPV = A/(A + B) × 100%, NPV = D/(C + D) × 100%. Accuracy of the test was calculated as (A + D)/(A + B + C + D) × 100%. The gold standard was conventional microbiological methods of bacteria identification described above.

The limit of detection was investigated using 10-fold serial dilutions of the total genomic DNA.

To determine required minimal LAMP reaction time, we examined the results of the reactions for *tox* and *dtxR* markers after 10, 20, 30, 40, 50, and 60 min of incubation using 10-fold serial dilutions of the total genomic DNA as a reaction template.

## Results

**Fig. 1:**
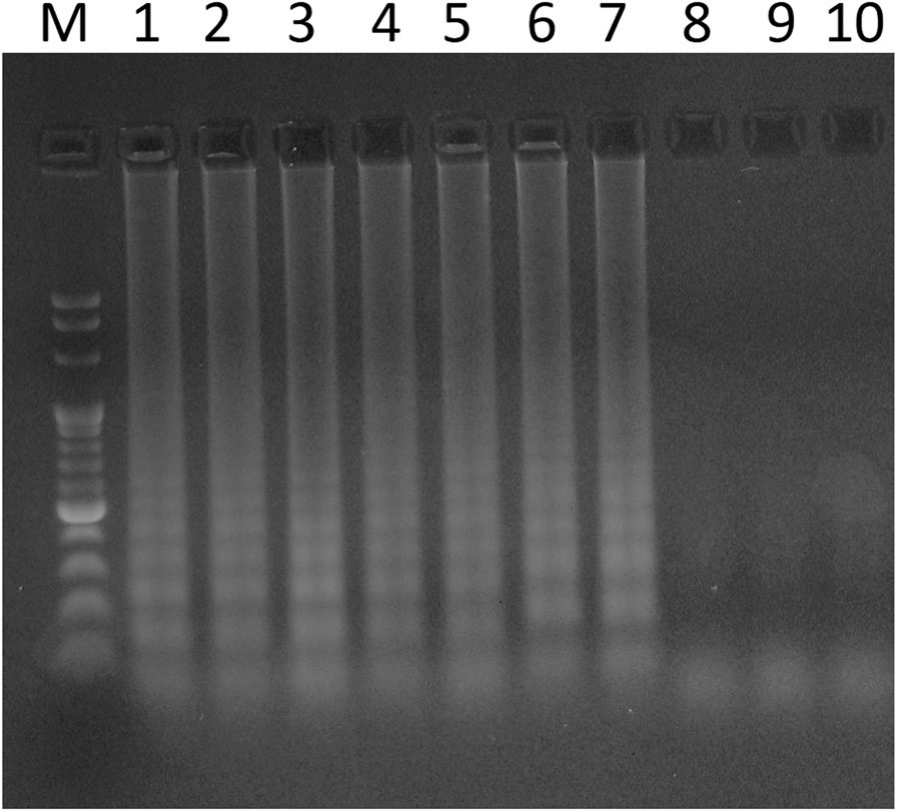
Agarose gel electrophoresis of products of the LAMP reaction for *tox* gene conducted for 60 min at various temperatures. M—Molecular Ladder, lane 1—incubation at 62 °C, lane 2—incubation at 64 °C, lane 3—incubation at 65 °C, lane 4—incubation at 66 °C, lane 5—incubation at 67 °C, lane 6—incubation at 68 °C, lane 7—incubation at 70 °C,and lanes 8–10—negative controls

The species-specific *dtxR* gene present in all *C. diphtheriae* strains and the *tox* gene present only in potentially toxigenic *C. diphtheriae, C. ulcerans*, and *C. pseudotuberculosis* strains were selected as target genes for designing the LAMP primers. Initially, three sets of primers for each of the genetic markers investigated were designed but only the sets presented in Table 1 did not yield false-positive results and therefore were selected for the study. The concentration of each of the primer as well as other reagents in the reaction mixture was optimized. Labeling of the primers FIB and BIP with biotin and FITC did not influence the amplification reaction, as it was assessed based on agarose gel electrophoresis results. The efficiency of the LAMP reaction was comparable in the temperature ranging from 62 °C to 70 °C (Fig. 1). For the study, we selected 65 °C as recommended by the manufacturer of the Bst 2.0 DNA polymerase.

**Fig. 2:**
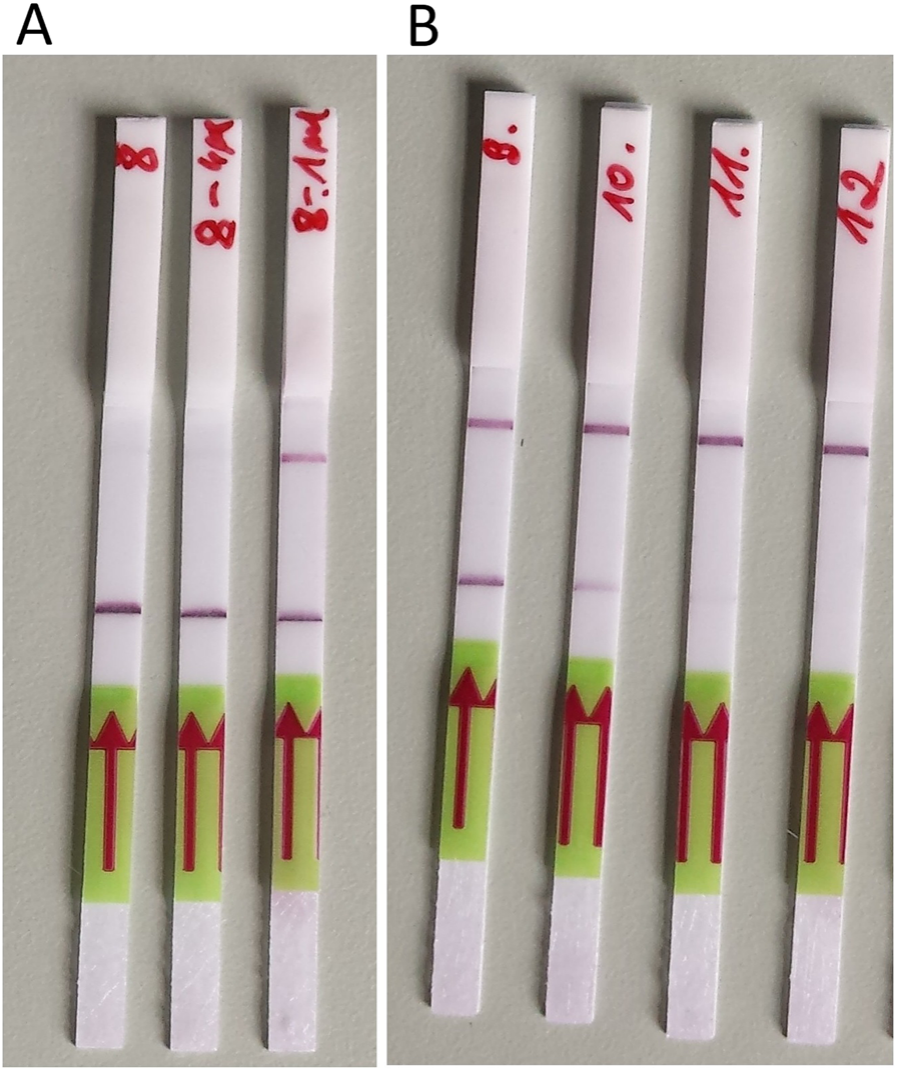
Visualization of LAMP for *dtxR* gene using lateral flow dipsticks. A lower band indicates positive results of the amplification. A higher band is a control of the lateral flow test. A high concentration of amplified products may cause lack of a control band. **a**—serial dilutions of the amplified product; **b**—serial dilutions of the DNA sample

We could detect positive LAMP reactions with the naked eye using HNB, calcein, QuantiFluor, and Milenia HybriDetect dipsticks. The positive reaction was clearly visible when the used HNB concentration was 0.125, 0.16, and 0.25 mM. For further studies, we selected the concentration 0.16 mM of HNB. The optimal amounts of calcein and QuatiFluor per reaction were 0.5 and 2 μl, respectively (Table 2). By using Milenia HybriDetect dipsticks, we observed atypical results for samples with a high concentration of DNA. According to manufacturer’s instructions, two color bands should be visible on the dipstick for positive results: test band and control band, whereas only control band should be visible for negative results. However, we observed that when the concentration of amplicons was high, the control band was not visible (Fig. 2). This issue was overcome by the dilution of the amplified product. We could not detect positive LAMP reaction when the neutral red and phenol red were used. It was probably because the pH changes during the reaction were very subtle.

**Table 2:** Comparison of the optimal concentration of various indicators for colorimetric detection of LAMP results

Indicatior	Concentration (mM)									
	2	1.5	1	0.5	0.32	0.25	0.16	0.125	0.08	0.04
HNB	–	nt	–	–	–	+	+	+	–	–
calcein	–	–	–	+	nt	–	nt	nt	nt	nt
QuantiFluor	+	nt	–	–	nt	nt	nt	nt	nt	nt

*nt* not tested

**Fig. 3:**
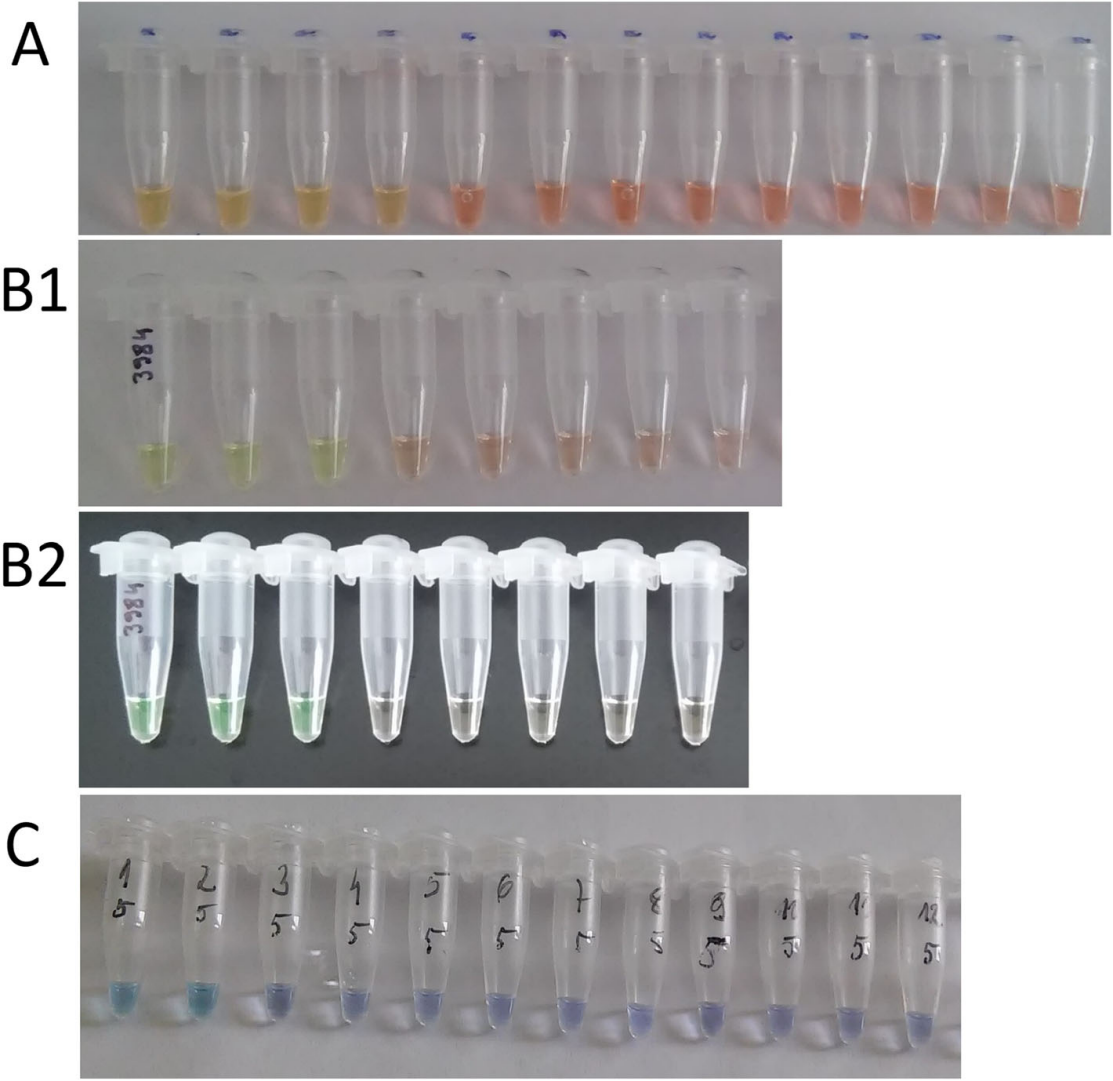
Results of the LAMP detection limit for *tox* gene. From the left to the right, 10-fold serial dilutions of the DNA samples. A—60 min of incubation, detection using QuantiFluor; B—50 min of incubation, detection using calcein (B1—white background, B2—black background); C—40 min of incubation, detection using HNB

The sensitivity and specificity of the LAMP reaction for *tox* and *dtxR* markers were comparable using HNB, calcein, and QuantiFluor, as well as Milenia HybriDetect dipsticks, and both were calculated as 100%. PPV, NPV and accuracy were also 100% (Table 3 and Table 4). The detection limit was also comparable for both genetic markers and all product detection methods and estimated as 1.42 pg/μl concentration of DNA template, which means 2.84 pg of DNA in 25 μl of the reaction mixture, when the reaction was conducted for 60 min. However, the detection limit lowered 10 times for every 10 min of reduction in the time of incubation during the reaction (Table 5, Fig. 3).

**Table 3:** Results of the LAMP for *tox* gene and *dtxR* gene for various DNA samples

Bacterial species (number of strains tested)	LAMP results	
	*tox*	*dtxR*
Toxigenic *Corynebacterium diphtheriae* (8)	+	+
Non-toxigenic *Corynebacterium diphtheriae* (31)	–	+
Toxigenic *Corynebacterium ulcerans* (1)	+	–
*Streptococcus salivarius* (1)	–	–
*Streptococcus pneumoniae* (1)	–	–
*Streptococcus pyogenes* (1)	–	–
*Streptococcus epidermidis* (1)	–	–
*Haemophilus influenzae* (1)	–	–
*Moraxella catharalis* (1)	–	–
*Staphylococcus aureus* (1)	–	–
*Corynebacterium pseudodiphtheriticum* (1)	–	–
*Pseudomonas aeruginosa* (1)	–	–
*Escherichia coli* (1)	–	–
*Klebsiella pneumoniae* (1)	–	–

**Table 4:** 2X2 contingency table for tests developed in the study. A – detection of *tox* gene, B – detection of *dtxR*

A		Gold standard		
		Positive	Negative	Measures
Test results	Positive	9	0	PPV 9/(9 + 0) × 100% = 100%
	Negative	0	42	NPV 42/(0 + 42) × 100% = 100%
	Measures	Sensitivity 9/(9 + 0) × 100% = 100%	Specificity 42/(0 + 42) × 100% = 100%	Accuracy (9 + 42)/(9 + 0 + 0 + 42) × 100% = 100%
B		Gold standard		
		Positive	Negative	Measures
Test results	Positive	39	0	PPV 39/(39 + 0) × 100% = 100%
	Negative	0	12	NPV 12/(0 + 12) × 100% = 100%
	Measures	Sensitivity 39/(39 + 0) × 100% = 100%	Specificity 12/(0 + 12) × 100% = 100%	Accuracy (39 + 12)/(39 + 0 + 0 + 12) = 100%

**Table 5:** Results of examination the minimal incubation time required for the LAMP assay when various concentrations of DNA template have been used

Incubation time (min)	DNA dilution (used as a template)					Negative control
	1.42 ng/μl	142 pg/μl	14.2 pg/μl	1.42 pg/μl	142 fg/μl	
10	–	–	–	–	–	–
20	–	–	–	–	–	–
30	+	–	–	–	–	–
40	+	+	–	–	–	–
50	+	+	+	–	–	–
60	+	+	+	+	–	–

## Discussion

Diphtheria is a vaccine-preventable disease. Currently, the diphtheria vaccination coverage varied from 42% in some developing countries to 99% in some developed countries [16]. Diphtheria outbreaks occur in endemic countries, and diphtheria cases are reported every year in EU/EEA. According to ECDC data, 216 diphtheria cases were reported in EU/EEA in the period 2012–2016. Most of them were imported from endemic geographical areas and some were indigenous cases [17]. Moreover, there have been many cases being described involving *C. diphtheriae* and *C. ulcerans*, which do not fit the WHO definition of diphtheria. These cases include serious invasive infection with high mortality rates caused by nontoxigenic strains of *C. diphtheriae* as well as cutaneous infections, such as non-healing ulcers, caused by toxigenic and non-toxigenic *C. diphtheriae* [10, 11, 17–19], whereas WHO diphtheria case definition cover only an illness of the upper respiratory tract characterized by the following: pharyngitis, nasopharyngitis, tonsillitis or laryngitis and adherent pseudomembrane of the pharynx, tonsils, larynx and/or nose [20].

Epidemiological data on diphtheria and a growing problem of nontoxigenic *C. diphtheriae* invasive infections have revealed the need for point-of-care testing (POCT) technology for the detection of *C. diphtheriae* in carriers, suspected cases, and contacted persons. Such POCT technology would be of great value especially in endemic regions of the disease, where access to health care is limited, and in refugee camps, to timely start appropriate treatment and further prevent the spread of the *C. diphtheriae* and the outbreak. In our study we developed simple and rapid testing methods based on LAMP assay for the detection of *C. diphtheriae* and differentiation between toxigenic and non-toxigenic strains. The developed method was highly sensitive and specific and showed a very low detection limit. It was reported by other researchers that LAMP detection limit is 100–1000 times lower than polymerase chain reaction (PCR) [21]. However, we found that the incubation time necessary to obtain positive results depends on the amount of the target DNA in the sample. The methods of visualization the results, including the use of HNB, QuantiFluor, calcein, and Milenia HybriDetect dipsticks, did not influence the detection limit, but colorimetric detection using HNB and calcein, which were added to the reaction mixture before incubation, are superior to QuantiFluor and Milenia HybriDetect dipsticks because they enable faster detection of positive reaction in the real-time mode, and no additional handling after reaction is needed. The opening tubes after the reaction is associated with an increased risk of contamination of other subsequent LAMP reaction solutions. On the other hand, differences in transparency of various plastic tubes might influence on readability of color change. Moreover, color interpretation can be subjective.

Regardless of the visualization method used, it must be kept in mind that a small percentage of strains are *tox* gene positive but do not produce active toxin [22, 23]. Therefore, strains positive for *tox* should subsequently be tested by the Elek test for expression of diphtheria toxin.

The LAMP reaction temperature ranging from 62 °C to 70 °C, as revealed in our study, shows that the heating device used does not have to be very precise. Hatano et al. [24] proposed the conduction of LAMP using a disposable pocket warmer placed in a Styrofoam box. It makes the LAMP assay independent from any electric power and therefore applicable in sites where electricity infrastructures are inadequate, such as undeveloped areas. LaBarre et al. [25] developed the non-instrumented nucleic acid amplification heater suitable for isothermal amplification methods, where heat is generated by an exothermic reaction of CaO with water. Cost of the LAMP assay proposed in our study is 2–25 euros per 50 samples tested (depending from the selected dye) when colorimetric visualization is used and about 120 euros per 50 samples when the dipstick visualization is used. The assay is fairly cost-effective when combined with colorimetric visualization with HNB and calcein. The QuantiFluor is more expensive and increases the cost of testing.

One of the disadvantages of most molecular methods is the requirement for storage conditions of reagents, such as polymerases, which usually have to be kept in freezing. However, a lyophilized mastermix containing all reagents required for LAMP assay was developed for the detection of some other pathogens [25, 26]. Furthermore, LAMP reagents are commercially available in a dry format currently, which can be stored at room temperature.

At the stage of development of the potential point-of-care test for diphtheria, we used DNA samples. However, according to Yan et al. [27] and Dugan et al. [28], the LAMP assay can be conducted directly from the bacterial colony, as high temperature causes the leak of bacterial cells, after which a high amount of DNA is released. Therefore, our test might be validated to be used for clinical samples directly. It was confirmed that LAMP assay was less affected by various components of clinical samples compared to other molecular methods, such as PCR [27, 29], and therefore, the DNA samples do not have to be purified perfectly. Though, the assays proposed in the study should be validated further using matrices which might contain inhibitors, such as sputum, and real clinical samples.

The limitation of the study is a limited number of non-target bacterial species tested. Based on the other studies and genome sequences available in GenBank the *tox* and *dtxR* markers are specific. Nevertheless, the proposed LAMP assay should be tested using more strains of other species, with a special focus on Corynebacterium genus.

## Conclusions

The developed LAMP for diphtheria diagnostics might be a valuable tool for outbreak investigations, especially in endemic areas, as well as for rapid screening of travelers coming from diphtheria-endemic regions. It can also be used for the examination of carriers and diphtheria contact persons. The selection of *dtxR* gene, apart from *tox* gene, as a diagnostic marker enables also the detection of non-toxigenic *C. diphtheriae* strains in tested samples, which is important in countries where nontoxigenic *C. diphtheriae* invasive infections are reported [10, 18].

The assay developed in the study has the potential to be integrated into a diagnostic mobile device for POCT of diphtheria and other *C. diphtheriae* infections, which will be highly sensitive, specific, inexpensive, easy to use, and applicable in low-resource settings. Although our study revealed high specificity and sensitivity and low limit detection of the assay, it should be tested using real clinical samples directly. Future endeavors would include applying these technologies to clinical materials.

## Data Availability

The datasets generated and/or analysed during the current study are available in the GenBank repository, accession number BX248353.

## Abbreviations

LAMP: Loop-Mediated Isothermal Amplification
HNB: Hydroxynaphthol blue
POCT: Point-of-care testing
EU/EEA: European Union/European Economic Area
